# Using a self-modulated treadmill as a novel approach to study cognitive-motor and biomechanical outcomes during dual-task walking in individuals with and without lower limb loss

**DOI:** 10.1007/s00221-025-07209-2

**Published:** 2026-01-21

**Authors:** Emma P. Shaw, Sarah R. Bass, Jonathan R. Gladish, Kyle Pietro, Alexandra A. Shaver, Christopher Gaskins, Steven Kahl, Christopher L. Dearth, Matthew W. Miller, Alison Pruziner, Bradley D. Hatfield, Brad D. Hendershot, Rodolphe J. Gentili

**Affiliations:** 1 Neuroscience and Cognitive Science Program, University of Maryland, College Park, MD USA; 2 Cognitive Motor Neuroscience Laboratory, Department of Kinesiology, School of Public Health, University of Maryland, Bldg #255, Room #2140, College Park, MD 20742 USA; 3 Department of Rehabilitation, Walter Reed National Military Medical Center, Bethesda, MD USA; 4 Department of Physical Medicine and Rehabilitation, Uniformed Services University of the Health Sciences, Bethesda, MD USA; 5 Extremity Trauma and Amputation Center of Excellence, Defense Health Agency, Falls Church, VA USA; 6 Department of Surgery, Uniformed Services University of the Health Sciences, Bethesda, MD USA; 7 School of Kinesiology, Auburn University, Auburn, AL USA; 8 Maryland Robotics Center, University of Maryland, College Park, MD USA; 9 Institute for Systems Research, University of Maryland, College Park, MD USA; 10 Artificial IntelligenceInterdisciplinary Institute, University of Maryland, College Park, MD USA

**Keywords:** Lower limb loss, Mental workload, Electroencephalography, Biomechanics, Self-paced walking, Dual-task walking

## Abstract

Combined examination of mental workload and biomechanics during dual-task walking in individuals with lower-limb loss is limited to fixed, but not self-modulated walking pace, for which the latter enables dynamic cognitive-motor behavior as typically engaged during community ambulation. By assessing electroencephalography (EEG) (theta, low/high-alpha power) and biomechanics (gait speed, double limb support, stride width), the cerebral cortical activity underlying mental workload and walking mechanics were examined when individuals with and without lower-limb loss executed a cognitive task (assessed via response time and accuracy) under variable demand (seated and walking). Both populations maintained walking mechanics (unchanged gait speed, double limb support, stride width) during dual-task walking across demand and exhibited similarly elevated neurocognitive engagement (e.g., attention, action monitoring) indicated by similar theta power increase and low/high-alpha power decrease when facing greater demand. However, injured individuals exhibited relative performance decrement (degraded response time/accuracy), which suggests attenuated cognitive-motor efficiency relative to uninjured (i.e., similar cortical activity across groups with degraded performance). Moreover, while uninjured individuals during dual-task walking could robustly engage neurocognitive processes to maintain walking mechanics and successfully attend to the concurrent cognitive task, those with lower-limb loss did not exhibit such a robust recruitment (i.e., unchanged frontal/temporal high-alpha power). Such alterations in individuals with lower-limb loss leads to maintenance of walking at the cost of a concurrent task. The present work informs rehabilitation practice and reveals specific cognitive-motor outcomes for individuals with lower-limb loss in an enhanced ecological context.

**Supplementary Information:**

The online version contains supplementary material available at 10.1007/s00221-025-07209-2.

## Introduction

Individuals with lower limb loss often describe walking with a prosthesis as cognitively demanding (Gauthier-Gagnon et al. 1999; Miller et al. 2001). Prior work has reported an overall elevation in cognitive effort among individuals with lower limb loss while walking, particularly with more proximal amputations (e.g., Pruziner et al. 2019 , Schack et al. 2022; Shaw et al. 2019) and possibly influenced by experience or prosthesis type (e.g., Aizu et al. 2022;; Marchand et al. 2021;fied cognitive effort observed among individuals with versus without lower limb loss may diminish capacity to allocate sufficient neural resources to ambulation demand, thereby reducing gait quality and/or attentiveness to the surrounding environment, ultimately increasing fall risk (Morgan et al. 2018; Omana et al. 2023). Indeed, individuals with versus without lower limb loss are 20% more likely to trip or fall, with over 50% of community-dwelling individuals with lower limb loss reporting a fall at least once per year (Miller et al. 2001). While falls may have detrimental repercussions in the near term, such as injuries and damage to prosthetic componentry (Hisano et al. 2020), individuals with lower limb loss can also become fearful after having fallen previously, leading to loss of independence, less community participation, and decreased socialization over the longer term (Miller et al. 2001). Dual-task paradigms are commonly used to examine the interactions between cognitive and motor processes, including individuals with and without lower limb loss (Morgan et al. 2018; Omana et al. 2023; Shaw et al. 2018, 2019b). However, methodological choices, such as selection of motor and cognitive tasks, directions for task prioritization, and inclusion of baseline (or single-task) conditions, collectively challenge the synthesis of results from these dual-task studies (Demirdel et al. 2022; Morgan et al. 2018). Importantly, while dual-task walking studies conducted on both a treadmill and overground report changing gait outcomes with a concurrent cognitive task (Dubost et al. 2006; Hollman et al. 2007; Yogev-Seligmann et al. 2008), particularly compared to fixed-speed treadmill walking, the ability to (continuously) self-modulate speed during dual-task walking can better represent community ambulation (Feasel et al., 2011; Kao et al., 2021; Sloot et al. 2014), whereby walking speeds can be modulated to adapt to the changing environmental requirements and task prioritization (e.g., without explicit prioritization instructions, uninjured individuals tend to prioritize walking to minimize risk of falling; Gerin-Lajoie et al. 2005; Schrodt et al. 2004; Yogev-Seligmann et al. 2012). Thus, self-regulated speed during dual-task walking enables a more flexible deployment of cognitive-motor processes underlying task planning and strategies (e.g., maintaining/reducing speed) compared to a fixed speed as imposed on individuals in much of the prior work (Yogev-Seligmann et al. 2012).

Building on our prior work with fixed-speed treadmill walking (i.e., Pruziner et al. 2019; Shaw et al. 2019), here we employed a dual-task paradigm with continuous self-modulation of walking speed to evaluate neurocognitive and biomechanical outcomes between individuals with versus without lower limb loss. In such a context, a prior framework suggested that the individual’s capacity to respond effectively to postural challenges would depend on their postural reserve (Gerin-Lajoie et al. 2005; Schrodt et al. 2004; Yogev-Seligmann et al. 2012). Thus, having a large postural reserve, uninjured individuals could successfully cope with elevated postural demand by limiting consumption of attentional resources that can, in turn, be redeployed to attend to any increasing concurrent task demands while maintaining walking mechanics and particularly speed (Yogev-Seligmann et al. 2012; Kao et al., 2021). Conversely, individuals with lower limb loss would likely be limited in postural reserve (i.e., requiring greater attentional resources for postural control) leading to altered walking mechanics. In turn, fewer attentional resources would be available to face an increasingly demanding concurrent cognitive task resulting in poorer performance compared to uninjured individuals. In such a context, the ability to self-pace locomotion during dual-task walking is important since it offers flexibility to prioritize specific strategies not necessarily possible when the locomotion pace is artificially fixed. For instance, injured individuals could deploy additional attentional resources to prioritize balance and maintain stable walking mechanics to avoid a potential fall or could voluntarily decrease their walking speed as a trade-off to share limited attentional resources between locomotion control and concurrent cognitive task performance. Therefore, the examination of both EEG-based cortical dynamics and biomechanics in such a context could reveal these mechanisms in individuals with and without limb loss and, more generally, extend the body of work that has examined changes in brain dynamics during self-paced dual-task walking (Huang et al. 2022; Pizzamiglio et al. 2017) limited to prefrontal regions while employing a fixed walking pace (Möller et al. 2020, 2024; Pruziner et al.^ 2019^; Shaw et al. 2018, 2019a).

Based on this theoretical framework, it was predicted that, as the concurrent task demands increase, uninjured individuals will maintain temporal-spatial walking characteristics, particularly speed, along with a fairly modest degradation of the secondary cognitive task performance achieved via an elevation of mental workload (indexed by an increase of theta power and a reduction of the low-alpha and high-alpha power). However, relative to uninjured individuals, those with lower limb loss will: (1) exhibit altered gait outcomes, as indicated by slower, more variable gait speed with wider stride widths and longer double limb support times, and (2) suffer limited attentional resources to attend to the secondary task leading to a greater elevation of mental workload (indexed by greater theta power and smaller low-/high-alpha power) and further degradation of the concurrent task performance. Finally, individuals with and without lower limb loss should similarly exhibit a decrement in task performance and an increase in mental workload as the cognitive task demand increases in the walking relative to the seated conditions.

## Methods

### Participants

Thirty-three participants, 14 with unilateral lower-limb loss (8 transtibial, 6 transfemoral) and 19 uninjured controls, provided informed consent to study procedures approved by the local Institutional Review Board and were screened for participation. After screening for orthopedic/neurological injury, psychotropic drugs and due to scheduling and technical issues the final sample consisted of 9 individuals with lower limb loss (6 transtibial and 3 transfemoral), and 12 uninjured controls. This study was limited to individuals with traumatic unilateral limb loss occurring at least six months prior. Additionally, all participants in the limb loss group were able to walk on a treadmill for five minutes at a time without the use of an assistive device other than their prosthesis. Participants were excluded from the study if they had a cardio-pulmonary, metabolic or integumentary diagnosis where walking for 15 min was contraindicated. All participants were screened to ensure the absence of brain injury that resulted in functional impairment, decreased learning capabilities, or the inability to follow complex commands (excluded via a Mini Mental State Examination score less than 23). Additionally, all participants were free of vestibular dysfunction and visual impairment that would detract from their ability to fully interact with the virtual reality environment. Pain was less than a 4 out of 10 on a visual analog scale at the time of study completion for all participants. No participant had a spinal cord injury that could have influenced their gait, and all participants were free from a skull deformity that prevented full contact of the EEG cap. Participants with limb loss did not have any contralateral injury that could have prevented full sagittal plane lower extremity motion required for gait on uneven terrain or contralateral ankle, knee or hip strength that impacted gait. Additionally, limb loss participants were free from heterotopic bone that limited functional range of motion or that resulted in pain that impeded performance. No participant had an upper limb amputation. Lastly, female participants were excluded if they were in their 2nd or 3rd trimester of pregnancy.

### Experimental protocol

Participants repeated two sets, one while seated and one while walking, each consisting of cognitive (N-back memory) tasks at three levels of demand: “low demand” (zero back (0B)), “medium demand” (one back (1B)), and “high demand” (two back (2B)). The presentation order of all six trials was individually randomized for each participant to minimize learning effects and acclimation to the tasks. Namely, random counterbalancing was employed in which every possible order of conditions was determined, and one was then randomly assigned to each participant. As a result, participants encountered a unique order of conditions. All tasks were up to 4 min in duration, performed in a virtual duck-hunting environment within the Computer Assisted Rehabilitation Environment (CAREN) system (Motekforce Link, Amsterdam, The Netherlands).

**Fig. 1:**
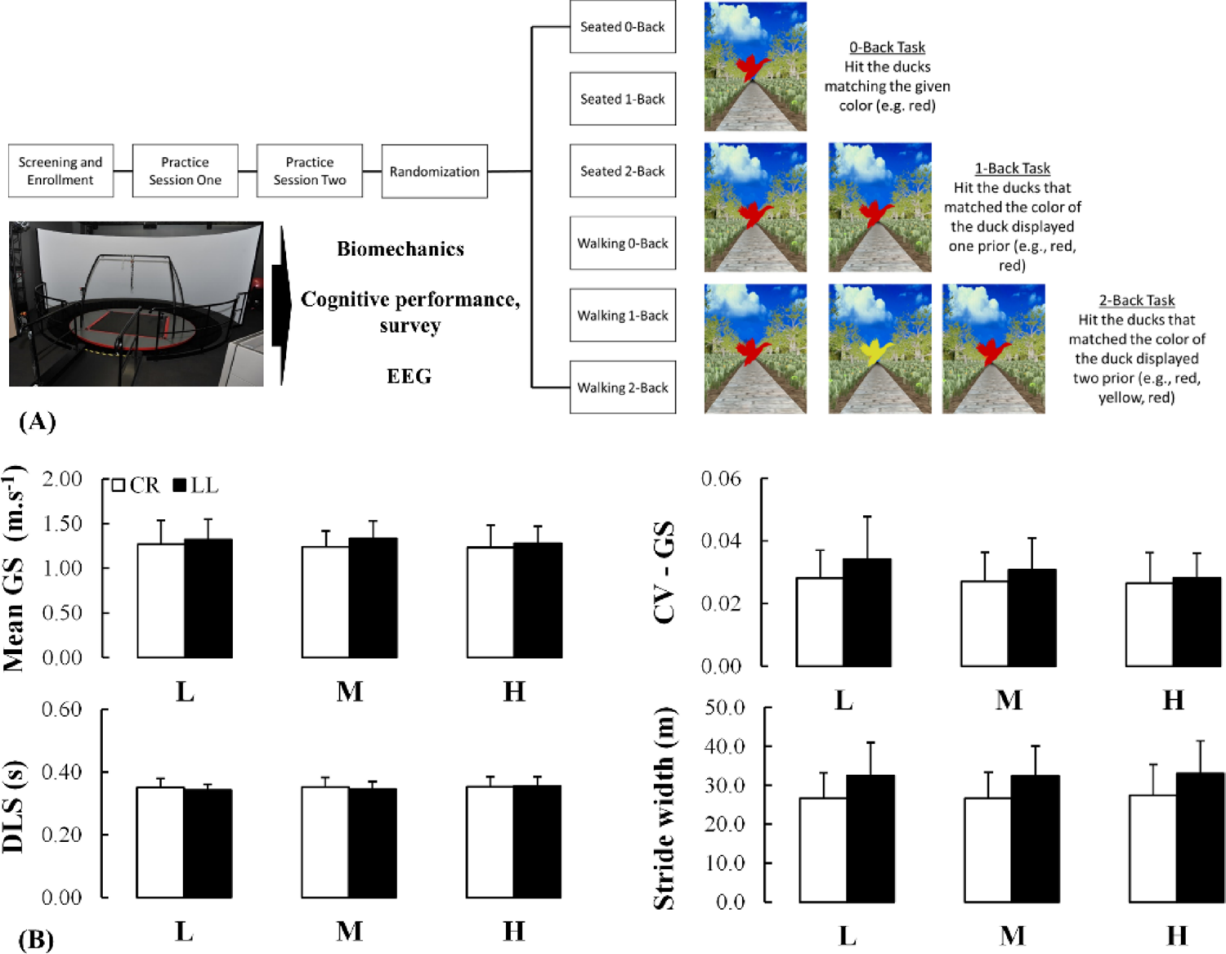
**A** Experimental set-up where individuals with and without lower limb loss executed the cognitive (N-back task) while being seated or walking on an instrumented treadmill within the CAREN system as biomechanics, cognitive performance, survey scores and EEG were collected. Visual description of the three N-back memory task demands: (1) zero-back [0B], (2) one-back [1B], and (3) two-back [2B]. **B** Spatio-temporal mechanics obtained for both uninjured (white bars) and injured (black bars) individuals while executing the cognitive task under varying demands during dual-task walking. CR: Control individuals without lower-limb loss, LL: Individuals with lower-limb loss, Mean GS: mean gait speed, CV GS: coefficient of variation in gait speed, DLS: double limb support time, SW: stride width, L: Low demand, M: Medium demand, H: High demand. *: *p* < 0.05; **: *p* < 0.01; ***: *p* < 0.001

All walking conditions employed a self-modulating treadmill speed algorithm within the CAREN system (Sloot et al. 2014), whereby the treadmill belt speed is modulated by changes in fore-aft position of the pelvis. During the seated conditions, a chair was placed on the right treadmill belt; the left treadmill belt was set to move at the participant’s self-selected speed to standardize the noise level generated by the treadmill belt and the speed at which the scene moved. Participants were asked to walk while completing the concurrent task (no explicit prioritization instructions were provided) in which participants were asked to hit a button on a handheld controller for ducks of matching colors. For the 0B, 1B and 2B task, participants were respectively asked to (1) only hit a duck matching a given color (e.g., red) (2) hit the ducks that matched the color of the duck displayed one prior (e.g., red, red) and (3) hit a duck that matched the color of the duck displayed two prior (e.g., red, yellow, red), respectively. The NASA-TLX was administered at the end of each of the trials to assess the participant’s self-reported physical and mental demands of the task (the other dimensions were assessed in an exploratory manner and as such were reported as supplementary material). After each task, participants were provided the opportunity to take a 2-min break to minimize the effects of fatigue. Prior to testing, participants completed two practice sessions during which they were introduced to the self-modulating treadmill and given the opportunity to test out the three different cognitive tasks (Fig. 1A).

### biomechanical data collection

During all walking conditions, lower limb kinematic data were continuously recorded by tracking (120 Hz) positions of 24 retro-reflective markers (modified Helen Hayes marker set) using a 12-camera optical motion capture system (Bonita T40 series, Vicon, Oxford, United Kingdom). Pelvic markers (i.e., anterior/posterior superior iliac spines) were affixed to a belt with the primary intention of ensuring adequate visibility for controlling the self-pacing treadmill algorithm and thus were placed approximately. Treadmill belt speed was continuously sampled (300 Hz) directly from the CAREN system (Motekforce Link, Amsterdam, The Netherlands).

### EEG data collection

As participants negotiated each cognitive-motor task, EEG was recorded from 64 scalp sites (extended 10–20 system) at a sampling frequency of 1000 Hz transmitted wirelessly through the MOVE system (Brain Products GmbH, Munich, Germany). All sensor impedances were maintained below 10 kΩ and band-pass filters were set at 0.01–100 Hz. Additionally, sensors were placed on the earlobes to serve as “non-brain” reference sites. The left earlobe served as an online reference and a common ground was employed at the FPz site on the scalp.

### Data analysis

#### Biomechanical data analysis

Raw marker trajectory data were filtered using a first-order, bidirectional, low-pass Butterworth filter with a cut-off frequency of 6 Hz and exported to Visual 3D (C-Motion, Germantown, MD, United States). Spatio-temporal measures were calculated including step/stride length, double limb support time, and stride width. Spatio-temporal variables were calculated according to heel-strike and toe-off events as identified by anteroposterior foot position with respect to the pelvis segment (Zeni et al. 2008). Gait speed was taken directly from the treadmill belt output within the CAREN system. Means, standard deviations, and coefficients of variation for all variables were calculated for each twenty-second interval for the final three min of each task (or less if the task was completed in less than three minutes). The primary biomechanical metrics of interest included mean gait speed, coefficient of variation of gait speed, double limb support time, and stride width.

#### Cognitive task performance and surveys

Task performance was measured by task accuracy and response time across all three levels of demand while seated and walking. Task accuracy was assessed across the entire trial taking into account correct and incorrect responses by employing the Eq. (1) below.

1 $$\:Task\:accuracy=\frac{\left(Correct\:duck\:hits+Correct\:duck\:ignore\right)}{Total\:ducks\:encountered}\:X\:100$$

Response time was assessed as the time elapsed between a duck appearing on the screen and response hit on the game controller by the participant. Response time was only calculated for correct duck hits. Additionally, the NASA-TLX scores were computed for all the six dimensions of the survey although the mental and physical demand dimensions were of primary interest since they are the most relevant to the mental workload and physical effort, respectively (Akizuki and Ohashi 2015; Kujala 2012; Shaw et al. 2018; Shuggi et al. 2019). The other dimensions were assessed in an exploratory manner and, as such, are reported in the supplementary material.

#### EEG data analysis

EEG data were first re-referenced to an averaged ears montage offline. Next, EEG data were low-pass filtered at 50 Hz with a 48-dB rolloff and notch filtered at 60 Hz using a zero-phase shift Butterworth filter offline. The pruning technique (Onton et al. 2006) was then employed and eye movement artifact was reduced by using the ICA-based ocular artifact rejection function embedded in the analyzer software. Electrode FP2 served as the VEOG channel and electrodes AF7 and AF8 served as the bipolar HEOG channel. The VEOG and HEOG algorithm identified ICA-derived components that accounted for 70% and 30% of the amount of variance in the entire signal, respectively. These components were then removed from the raw EEG signal and the EEG signal was reconstructed for further processing. Following the completion of ICA-based ocular artifact rejection, data were epoched into 1 s sweeps and baseline corrected using the mean potential (0–1000 ms). Visual inspection of the epochs was performed to remove any remaining artifact and epochs that were too noisy were rejected. Then, to assess to what extent the EEG quality was affected by walking in absence (i.e., uninjured) and presence of transtibial (i.e., injured transtibial) as well as transfemoral (i.e., injured transfemoral) prostheses, the number of cleaned epochs kept was examined. Namely, a series of Wilcoxon signed rank tests (non-parametric tests were used due to small sample sizes) were employed to compare the number of cleaned epochs for seating versus walking for the three subgroups (i.e., uninjured, injured transtibial and injured transfemoral participants). Also, a series of Wilcoxon rank sum tests were used to compare the number of cleaned epochs between the uninjured, injured transtibial and injured transfemoral participants during walking. To account for multiple comparisons, a false discovery rate correction was employed. The Cliff (*δ)* effect size (due to the small sample size of each injured subgroup) was computed for all the contrasts. The results revealed that: (1) while smaller, the number of kept epochs for seated versus walking were comparable for the uninjured, injured transtibial and injured transfemoral participants (*p* ≥ 0.156); (2) the number of kept epochs for seated vs. walking revealed a small, small-to-moderate and moderate effect size for this contrast for the uninjured (*p* = 0.156, δ = 0.167), injured transtibial (*p* = 0.157, δ = 0.389) and injured transfemoral (*p* = 0.750, δ = 0.556) participants, respectively; (3) although lower for both injured subgroups relative to uninjured individuals, the number of kept epochs did not significantly differ between the groups during walking (*p* ≥ 0.750; δ ≤ 0.278). Therefore, although not statistically significant, there was a decrease in the number of epochs kept from seated to walking for all participants after EEG denoising, with such a reduction being more pronounced with injured participants and particularly those using a larger device due to a higher level of lower limb loss. Overall, these findings suggest that the quality of the EEG signal during walking vs. seated was not significantly degraded for all participants, although it was somewhat further altered for injured individuals and particularly for those operating a transfemoral prosthesis. Lastly, using these cleaned epochs, spectral power was calculated across 1-Hz bins and summed across the frequency bandwidths theta (4–7 Hz), low-alpha (8–10 Hz) and high-alpha (11–13 Hz) for the evaluation of mental workload (Gentili et al. 2018; Shaw et al. 2019a, b; Whittier et al. 2020). Spectral power for each frequency bandwidth was normalized by the spectral power of the entire spectrum considered to account for potential differences in brain dynamics between the groups of participants. Spectral values for each bandwidth were analyzed and compared across the frontal (F3, F4), central (C3, C4), temporal (T7, T8), parietal (P3, P4) and occipital (O1, O2) regions. The frontal theta/parietal alpha (FT/PA) and frontal theta/frontal alpha (FT/FA) ratio power were also computed as they have been previously shown to serve as robust indices of changes in mental workload (Gentili et al. 2018; Gevins and Smith 2003; Shaw et al. 2018, 2019a, 2019b).

### Statistical analysis

The variables of mean gait speed, coefficient of variation in gait speed, double limb support time, and stride width were analyzed by employing a series of 2 Group (Uninjured vs. Limb Loss) × 3 Demand (0B vs. 1B vs. 2B) mixed-model ANOVAs with Demand and Group represented as within- and between-subjects factors, respectively. The overall accuracy and response time for the cognitive task performance as well as the NASA-TLX scores and both (FT/FA, FT/PA) power ratios were analyzed by conducting a series of 2 Group (Uninjured vs. Limb Loss) × 2 Condition (Seated vs. Walking) × 3 Demand (Low vs. Medium vs. High)] mixed-model ANOVAs within which Demand and Condition represented within-subject factors while Group was a between-subjects factor. The changes in theta power, low-alpha and high-alpha power were examined by conducting a series of 2 Group (Uninjured vs. Limb Loss) × 2 Condition (Seated vs. Walking) × 3 Demand (Low vs. Medium vs. High) × 2 Hemisphere (Left vs. Right) × 5 Region (Frontal, Central, Temporal, Parietal, Occipital) mixed-model ANOVAs within which Condition, Demand, Hemisphere, and Region represented within-subjects factors and Group was a between-subjects factor. For all analyses, the Greenhouse-Geisser correction was utilized when the assumption of sphericity was not met. Whenever appropriate, post-hoc analyses were conducted by means of a Tukey post-hoc test while partial eta square and Cohen’s effect size were also reported. The significance level for all analyses was set at *p* < 0.05. An exploratory analysis was also conducted to examine if the findings related to the contrasts of primary interest obtained here involving individuals with limb loss while dual-task walking appeared to be driven by the level of limb loss (i.e., transtibial (*n* = 6) vs. transfemoral (*n* = 3)). Considering the analysis for several metrics and condition and the small sample size of the patients, only descriptive statistics were employed. Namely, the median, lower/higher quartile along with Cliff (*δ)* effect size (due to the small sample size of each injured subgroup) were computed and represented graphically along with the individual data points (see supplementary material for details).

## Results

### Biomechanics

There were no significant main or interactive effects for mean gait speed (*p* > 0.431), coefficient of variation in gait speed (*p* > 0.067), double limb support time (*p* > 0.093), or stride width (*p* > 0.062; Fig. 1B). Despite no statistical difference, the descriptive statistical exploratory analysis revealed that the median CV (and individual data points) was larger for individuals with transtibial vs. transfemoral lower limb loss, however the corresponding effect sizes were small (0.110 < *δ* < 0.334 for all demands). For all demands, the stride width was smaller for individuals with transtibial vs. transfemoral limb loss (0.556 ≤ *δ* ≤ 0.667 for all demands) (see supplementary material for details).

### Secondary cognitive task

#### Overall accuracy

**Fig. 2:**
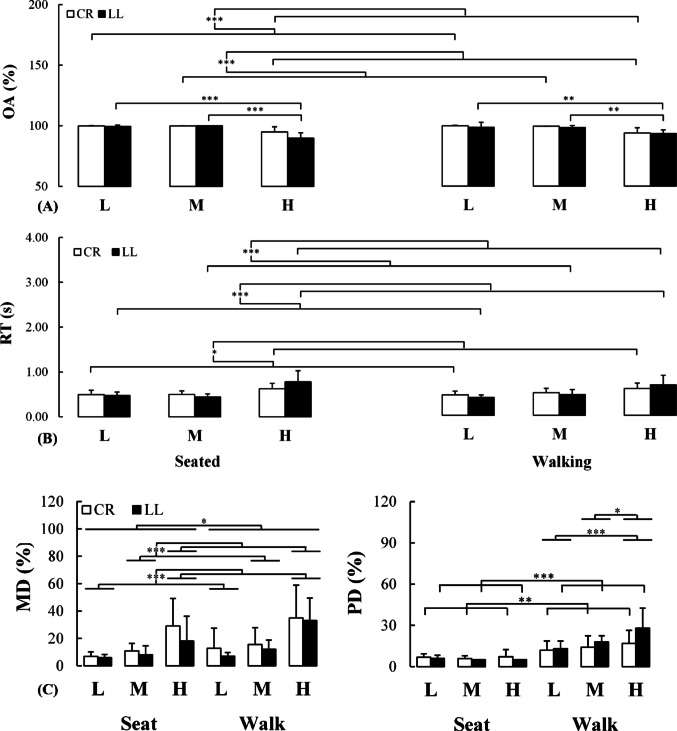
Changes in cognitive task performance (**A,B**) as well as mental and physical demand (**C**) for both uninjured (white bars) and injured (black bars) individuals while executing the cognitive task under varying demands while being seated or during dual-task walking. CR: Control individuals without lower-limb loss, LL: Individuals with lower-limb loss, L: Low demand, M: Medium demand, H: High demand. RT: Response time, OA: Overall accuracy, MD: Mental demand, PD: Physical demand. *: *p* < 0.05; **: *p* < 0.01; ***: *p* < 0.001

For the overall task accuracy a Group x Condition x Demand interaction effect (F(1.925, 32.729) = 6.656, *p* = 0.004, η_P_^2^ = 0.281) was observed. To further investigate this interaction, a Condition x Demand ANOVA was conducted separately for each group. For the uninjured group, there was a main effect of Demand (F(2, 20) = 41.270, *p* < 0.001, η_P_^2^ = 0.805); the overall accuracy was reduced from the low to high demand (*p* < 0.001; d = 2.419) and from the medium to high demand (*p* < 0.001; d = 2.310). For the lower-limb loss group, a Condition x Demand interaction (F(2, 14) = 5.629, *p* = 0.016, η_P_^2^ = 0.446) was also detected. For both conditions, the overall accuracy was reduced from a low to high demand (Seated: *p* < 0.001; d = 3.033; Walking: *p* = 0.007; d = 1.401) and slightly further attenuated from a medium to high demand (Seated: *p* < 0.001; d = 3.282; Walking: *p* = 0.007; d = 2.129). No other results reached the significance level (Fig. 2A). The exploratory analysis revealed that individuals with transtibial and transfemoral lower limb loss both exhibited a reduction of the overall task accuracy from seated to walking under the three levels of cognitive demand (0.200 ≤ *δ* ≤ 0.333) (see supplementary material for details).

#### Response time

For the response time a Group x Demand interaction (F(2, 34) = 34.328, *p* = 0.008, η_P_^2^ = 0.246) and a Condition x Demand interaction (F(2, 34) = 4.309, *p* = 0.021, η_P_^2^ = 0.202) were detected; while an elevation of the response time was observed from the low to high demand for both groups (Uninjured: *p* = 0.011, d = 1.202; Limb Loss: *p* < 0.001, d = 1.709), only the lower limb loss group increased response time from the medium to high demand (*p* < 0.001, d = 1.588) (Fig. 2B). Meanwhile, for both conditions, the response time increased from the low to high demand (Seat: *p* < 0.001, d = 1.360; Walking: *p* < 0.001, d = 1.527) and from the medium to high demand (Seat: *p* < 0.001, d = 1.455; Walking: *p* < 0.001, d = 1.035) (not shown in Fig. 2B). No other results reached the significance level (*p* > 0.05)  ). The exploratory analysis revealed that individuals with transtibial and transfemoral lower limb loss both increased their response time from seated to the walking condition under the three cognitive demands (0.067 ≤ *δ* ≤ 0.733 for all demands) (see supplementary material for details).

### NASA-TLX survey

The perception of mental demand for both groups increased from seated to the walking condition (F(1, 14) = 6.914, *p* = 0.020, η_P_^2^ = 0.331). Also, a main effect of task Demand was detected across groups (F(1.127, 15.783) = 15.817, *p* < 0.001, η_P_^2^ = 0.530); perceived mental demand increased from low to high (*p* < 0.001, d = 1.331) and medium to high demand (*p* < 0.001, d = 1.123). Also, for the perception of the physical demand, a Group x Condition interaction (F(1, 14) = 5.277, *p* = 0.038, η_P_^2^ = 0.404) was revealed. Post-hoc analysis revealed that the perceived physical demand was greater for the walking compared to the seated condition for both groups (Uninjured: *p* = 0.002, d = 1.191; Limb Loss: *p* < 0.001, d = 1.869). For this dimension a Condition x Demand interaction (F(2, 28) = 9.047, *p* < 0.001, η_P_^2^ = 0.393) was observed; an elevation of the concurrent task demand during walking resulted in participants perceiving the task as being more physically challenging (low vs. high: *p* < 0.001, d = 0.845; medium vs. high: *p* = 0.040, d = 0.500) whereas not while seated (*p* > 0.05) (Fig. 2C) (For the results for the other survey dimension see supplementary material for details).

### EEG

#### Theta

**Fig. 3:**
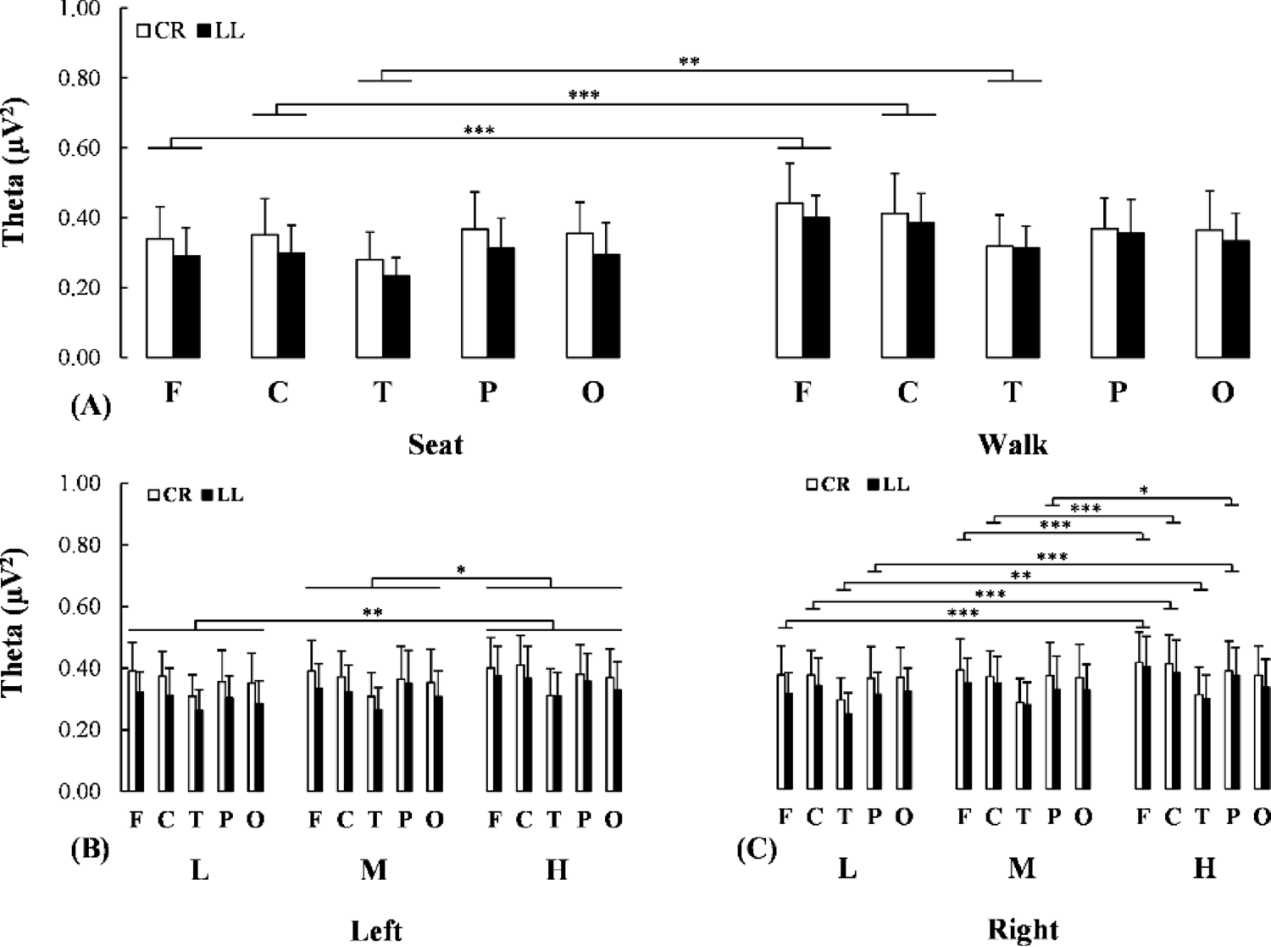
**A** Changes in theta power in the five scalp regions of interest for both uninjured (white bars) and injured (black bars) individuals while executing the cognitive task during seated or dual-task walking. **B,C** Changes in theta power in the left and right hemisphere for both uninjured (white bars) and injured (black bars) individuals while executing the cognitive task under varying demands for both task executions. CR: Control individuals without lower limb loss, LL: Individuals with lower limb loss, L: Low demand, M: Medium demand, H: High demand. F: Frontal, C: Central, T: Temporal, P: Parietal, O: Occipital. *: *p* < 0.05; **: *p* < 0.01; ***: *p* < 0.001

For the theta power a Condition x Region (F(2.807, 53.334) = 5.830, *p* = 0.002, η_P_^2^ = 0.235) interaction effect was detected; there was an elevation of theta power in the frontal (*p* < 0.001, d = 0.982), central (*p* < 0.001, d = 0.661) and temporal (*p* = 0.008, d = 0.578) regions when the task was executed while walking compared to seated (Fig. 3A). The exploratory analysis revealed a comparable elevation of theta power in the frontal, central and temporal regions for both individuals with transtibial and transfemoral limb loss (0.222 ≤ *δ* ≤ 0.333 for all demands) (see supplementary material for details).

For the same bandwidth a Demand x Region x Hemisphere (F(4.711, 89.503) = 2.727, *p* = 0.027, η_P_^2^ = 0.125) interaction effect was revealed. To examine this interaction separate Demand x Region ANOVAs were conducted on each hemisphere. For the left hemisphere, there was a main effect of Demand (F(2, 40) = 7.559, *p* = 0.002, η_P_^2^ = 0.274); an elevation of the level of task demand led to an elevation of theta power (low vs. high: *p* = 0.002, d = 0.293; medium vs. high: *p* = 0.026, d = 0.201) (Fig. 3B). For the right hemisphere, a Demand x Region interaction effect (F(8, 160) = 3.005, *p* = 0.004, η_P_^2^ = 0.131) was identified; an elevation of the level of task demand led to an elevation of theta power in the frontal (low vs. high: *p* < 0.001, d = 0.479; medium vs. high: *p* < 0.001, d = 0.297), central (low vs. high: *p* < 0.001, d = 0.319; medium vs. high: *p* < 0.001, d = 0.329), temporal (low vs. high: *p* = 0.008, d = 0.299) and parietal (low vs. high.: *p* < 0.001, d = 0.392; medium vs. high: *p* = 0.034, d = 0.271) regions (Fig. 3C).

#### Low-alpha

**Fig. 4:**
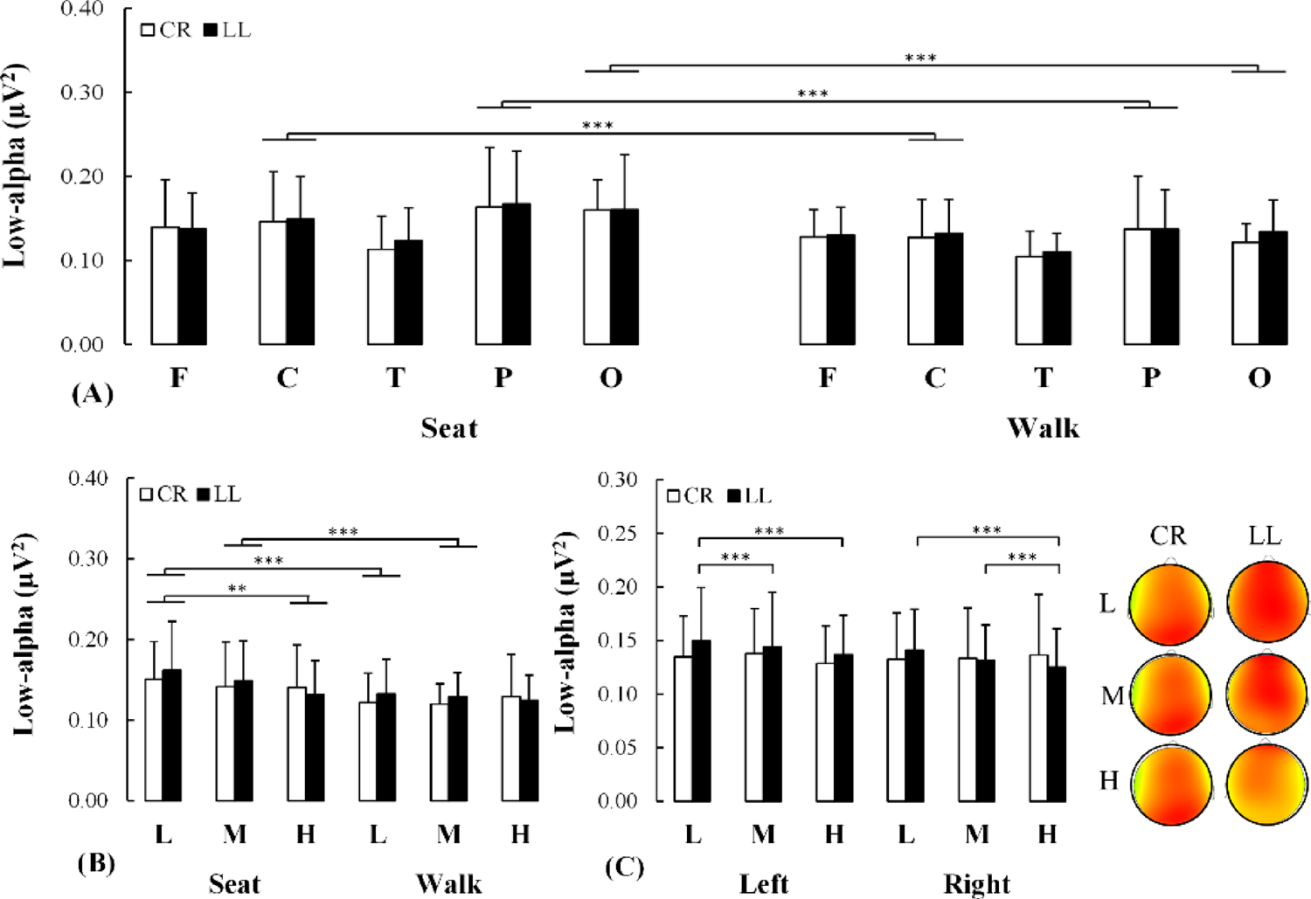
**A** Changes in low-alpha power in the five scalp regions of interest for both uninjured (white bars) and injured (black bars) individuals while executing the cognitive task during seated or dual-task walking. **B** Changes in low-alpha power for both individuals without lower-limb loss (white bars) and with lower-limb loss (black bars) while executing the cognitive task under varying demand while being seated or dual-task walking. **C** Changes in low-alpha power in the right and left hemisphere for both individuals without lower-limb loss (white bars) and with lower-limb loss (black bars) individuals while executing the cognitive task under varying demands for both task executions. On the right side of this panel, these changes in low-alpha power are quantitatively represented by a topographic scalp distribution. CR: Control individuals without lower-limb loss, LL: Individuals with lower-limb loss, L: Low demand, M: Medium demand, H: High demand. F: Frontal, C: Central, T: Temporal, P: Parietal, O: Occipital. *: *p* < 0.05; **: *p* < 0.01; ***: *p* < 0.001

For the low-alpha power, Condition x Region (F(2.701, 51.319) = 7.077, *p* < 0.001, η_P_^2^ = 0.271) and Condition x Demand (F(1.527, 29.012) = 4.453, *p* = 0.029, η_P_^2^ = 0.190) interaction effects were identified. For Condition x Region, there was an attenuation of low-alpha power in the central (*p* < 0.001, d = 0.333), parietal (*p* < 0.001, d = 0.429) and occipital (*p* < 0.001, d = 0.763) regions during dual-task walking compared to the seated condition (Fig. 4A). Also, the post-hoc analysis to examine the Condition x Demand interaction revealed an attenuation of low-alpha power for the walking relative to the seated condition for low (*p* < 0.001, d = 0.533) and medium (*p* < 0.001, d = 0.432) demand whereas this was not observed for the high demand (*p* = 0.319, d = 0.183). There was a reduction of low-alpha power from low to high demand for the seated but not walking conditions (*p* = 0.004, d = 0.321) (Fig. 4B). The exploratory analysis revealed a comparable level of low-alpha power in the central, parietal and occipital regions for both individuals with transtibial and transfemoral limb loss (0.222 ≤ *δ* ≤ 0.444 for all demands) (see supplementary material for details).

In addition, there was a Group x Demand x Hemisphere (F(1.688, 32.075) = 4.381, *p* = 0.019, η_P_^2^ = 0.203) interaction. To further examine this interaction, a Demand x Hemisphere ANOVA was conducted separately for the uninjured and the lower limb loss group. Although no significant findings were detected for the uninjured group, a Demand x Hemisphere (F(2, 16) = 6.920, *p* = 0.007, η_P_^2^ = 0.464) interaction effect was identified for the lower limb loss group. For the left hemisphere, there was an attenuation of low-alpha power from low to medium demand (*p* < 0.001, d = 0.236) and from a low to a high demand (*p* < 0.001, d = 0.356). However, for the right hemisphere, there was an attenuation of low-alpha power from low to high demand (*p* < 0.001, d = 0.364) and from medium to high demand (*p* < 0.001, d = 0.350) (Fig. 4C).

#### High-alpha

**Fig. 5:**
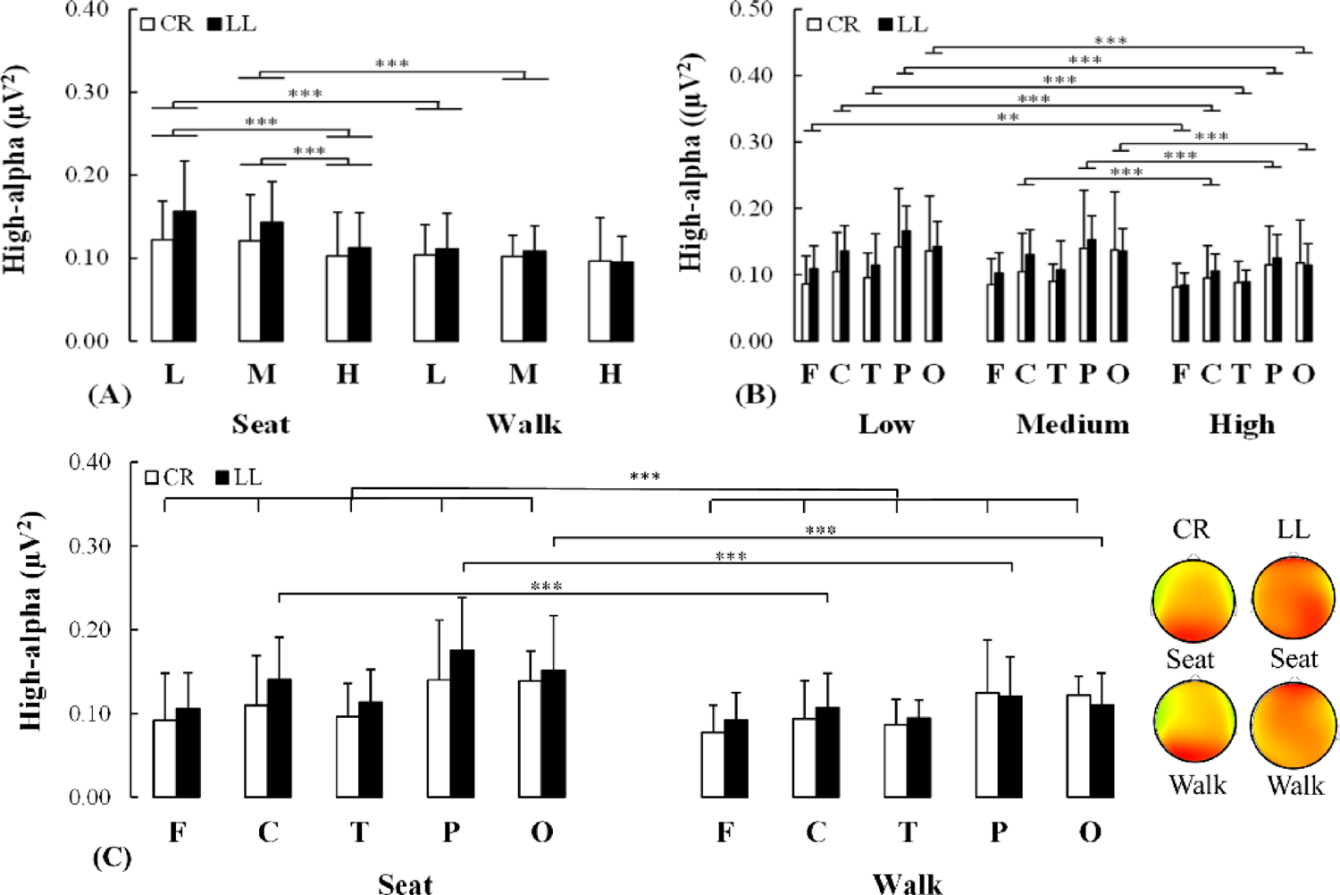
**A** Changes in high-alpha power for both uninjured (white bars) and injured (black bars) individuals while executing the cognitive task under varying demand while being seated or dual-task walking. **B** Changes in high-alpha power in the five scalp regions of interest for both uninjured (white bars) and injured (black bars) individuals while executing the cognitive task under varying demands for both task executions. **C** Changes in high-alpha power in the five scalp regions of interest for both individuals without lower-limb loss (white bars) and with lower-limb loss (black bars) while executing the cognitive task during seated or dual-task walking. On the right side of this panel, these changes in high-alpha power are qualitatively represented by a topographic scalp distribution. CR: Control individuals without lower-limb loss, LL: Individuals with lower-limb loss, L: Low demand, M: Medium demand, H: High demand. F: Frontal, C: Central, T: Temporal, P: Parietal, O: Occipital. *: *p* < 0.05; **: *p* < 0.01; ***: *p* < 0.001

For the high-alpha power Condition x Demand (F(1.646, 31.274) = 6.752, *p* = 0.006, η_P_^2^ = 0.262) and Demand x Region (F(3.837, 72.912) = 3.583, *p* = 0.011, η_P_^2^ = 0.159) interactions were detected. For the Condition x Demand interaction, there was a reduction of high-alpha power from low to high demand (*p* < 0.001, d = 0.526) and from medium to high demand (*p* < 0.001, d = 0.393) for the seated condition whereas this was not observed while walking. Also, a reduction of high-alpha power was observed from the seated to the walking condition for the low (*p* < 0.001, d = 0.482) and medium (*p* < 0.001, d = 0.412) but not high demand (Fig. 5A). For Demand x Region interaction, there was a reduction of high-alpha power in the frontal (*p* = 0.002, d = 0.354), central (*p* < 0.001, d = 0.356), temporal (*p* < 0.001, d = 0.373), parietal (*p* < 0.001, d = 0.501), and occipital (*p* < 0.001, d = 0.354) regions from low to high demand. Also, reductions of high-alpha power in central (*p* < 0.001, d = 0.314), parietal (*p* < 0.001, d = 0.395) and occipital (*p* < 0.001, d = 0.314) regions were observed from medium to high demand (Fig. 5B).

Furthermore, there was a Group x Condition x Region (F(2.536, 48.181) = 3.468, *p* = 0.029, η_P_^2^ = 0.154) interaction effect. To examine this interaction, a Condition x Region ANOVA was conducted separately for the uninjured and the lower limb loss group. For the uninjured group, the analysis only revealed a main effect of Condition (F(1, 11) = 20.043, *p* < 0.001, η_P_^2^ = 0.646), such that an attenuated high-alpha power was observed for the walking compared to the seated condition. For the lower-limb loss group, a Condition x Region interaction effect (F(4, 32) = 7.282, *p* < 0.001, η_P_^2^ = 0.477) was detected. The post-hoc analysis conducted to examine this Condition x Region interaction revealed an attenuation of high-alpha power in the central (*p* < 0.001, d = 0.792), parietal (*p* < 0.001, d = 1.156) and occipital (*p* < 0.001, d = 1.080) regions during walking compared to seated conditions (Fig. 5C). The exploratory analysis revealed a comparable reduction of high-alpha power in the central, parietal and occipital regions for both individuals with transtibial and transfemoral limb loss, although the effect size indicated that this modulation was more pronounced in the latter compared to the former (0.556 ≤ *δ* ≤ 0.778 for all demands) (see supplementary material for details).

#### Power ratios

**Fig. 6:**
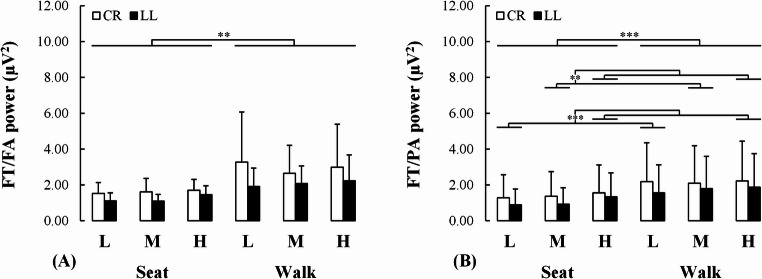
Changes in the frontal theta/frontal alpha ratio (FT/FA; panel **A**) and the frontal theta/parietal alpha ratio (FT/PA; panel **B**) power for both uninjured (white bars) and injured (black bars) individuals while executing the cognitive task under varying demand while being seated or dual-task walking. CR: Control individuals without lower limb loss, LL: Individuals with lower limb loss, L: Low demand, M: Medium demand, H: High demand. *: *p* < 0.05; **: *p* < 0.01; ***: *p* < 0.001

The FT/FA power ratio increased from seated to walking conditions (F(1, 19) = 37.504, *p* = 0.007, η_P_^2^ = 0.325). Similarly, the FT/PA power ratio increased from seated to walking conditions (F(1, 19) = 28.460, *p* < 0.001, η_P_^2^ = 0.600) (Fig. 6A). Lastly, the FT/PA power ratio increased as task demand became elevated (F(2, 38) = 11.536, *p* < 0.001, η_P_^2^ = 0.378); the FT/PA power ratio increased from low to high (*p* < 0.001, d = 0.307) and from medium to high (*p* = 0.005, d = 0.237) demand (Fig. 6B). The exploratory analysis revealed that individuals with transtibial versus transfemoral lower limb loss increased their median FTFA (0.556 ≤ *δ* ≤ 0.889) and FTPA (0.01 ≤ *δ* ≤ 0.333) power ratio from seated to walking, although this was more marked for the FTFA power ratio for the former relative to the latter patient subgroup (see supplementary material for details).

## Discussion

Despite biomechanical and neurocognitive similarities between groups, the specific differences observed here suggest that, as concurrent cognitive demands increased, those with vs. without lower-limb loss revealed a reduction of cognitive-motor efficiency (resulting from a similar/poorer performance being produced with greater brain resource engagement ) and an inability to robustly engage cognitive-motor resources (e.g., attention, working memory) during dual-task walking. First, the groups revealed similarities such that greater concurrent cognitive demands led to (1) the same walking pace and mechanics, and (2) an elevation of mental workload for both groups (as indexed by NASA-TLX scores, greater theta power and FT/PA power ratio, attenuated high-alpha power). Also, regardless of the concurrent demands, both groups revealed heightened mental workload proceeding from the seated to walking conditions. Finally, both groups revealed a similar reduction of low-/high- alpha power mainly for the seated and to a lesser extent for the walking conditions, as the concurrent cognitive demands increased. However, specific differences between groups were also identified. First, the response time remained unchanged for individuals with an intact limb as the cognitive demand increased from medium to high, whereas it increased for those with a lower-limb loss (Figs. 2A, B). Consistently, for the same medium to high contrast, the low-alpha power in the right hemisphere remained unchanged for the uninjured individuals whereas it was attenuated among individuals with lower-limb loss (Fig. 4C) indicative of increased effort in the latter group. Lastly, individuals without lower-limb loss revealed a whole-scalp attenuation of the high-alpha power from the seated to walking conditions, independent of the concurrent task demand, whereas such a modulation was restricted to the central, parietal and occipital regions for individuals with lower limb loss (Fig. 5C).

### Simultaneous changes in cognitive performance, mental workload and biomechanics common to both individuals with and without limb loss

#### Effect of the cognitive task demands

 Both groups maintained walking pace and gait biomechanics across the various concurrent cognitive task demands. The exploratory analysis revealed individuals with transfemoral versus transtibial lower limb may have a greater stride width, which is likely used to help maintain walking stability since their balance is more challenged by further compromised limb biomechanics (Hak et al. 2013; Jarvis et al. 2017; Morgan et al. 2016; Pruziner et al.^ 2019^ ). However, for both groups, as the cognitive demand increased, performance decrements accompanied greater levels of perceived mental workload for both seated and walking conditions. Furthermore, both groups similarly revealed theta synchrony across the entire left hemisphere but only in the right frontal, central and parietal regions as the cognitive task demands increased from low/medium to high demand. This agrees with the notion that increased theta power reflects an enhanced recruitment of cognitive resources to face higher cognitive demand. This increase of frontal, parietal and to a certain extent central theta power likely reflects greater engagement of the neural processing resources such as working memory, attentional and action monitoring when facing enhanced cognitive task demands (Cavanagh and Franck ., 2014; Coombes et al. 2005; Gevins and Smith 2003; Pruziner et al. 2019 ; Shaw et al. 2019a; Slobounov et al. 2013, 2015). Also, several prior studies have suggested that elevated central theta power reflects control of conflicting (i.e., dual-task) situations (Dini et al. 2022; Morís et al. 2018; Ma et al. 2015; Pan et al. 2020). Thus, the heightened cognitive task demands possibly engaged such a mechanism to identify the target stimulus when a more complex combination of stimuli was presented.

Besides theta modulations, both groups exhibited a reduction of high-alpha power for all cortical regions from low to high demand but limited to the central, parietal and occipital regions from the medium to high demand. This suggests a broad recruitment of task-specific resources to face greater cognitive demands. Since it is well established that the frontal, parietal and temporal regions hold a critical role in working memory and attentional control, such cortical dynamics suggest that they increasingly become involved as the cognitive demand increased (LaBar et al. 1999; Kardan et al. 2022; Pollmann et al. 2022). Also, greater engagement of the central and occipital regions would suggest enhanced recruitment of motor control processes and visual search related to cognitive control as task demands increase (Liegel et al. 2022; van den Berg et al. 2014). This agrees with the observed elevation of the FT/PA power ratio as the cognitive demand increases from low/medium to high task demand for both groups as well as with the well-established notion that when elevated, this power ratio reflects heightened mental workload (Gevins et al. 1997; Gevins and Smith 2003; Pruziner et al.^ 2019 ^; Shaw et al. 2019a).

Collectively, these EEG findings suggest that an elevation of the secondary task demands led both groups to increase the recruitment of their cognitive-motor processes, albeit producing poorer performance, thus leading to reduced cognitive-motor efficiency (Hatfield et al. 2020; Gentili et al. 2018).

#### Effect of task execution conditions (seated vs. dual-task walking)

Frontal, central and temporal theta power increased independently of the cognitive demands during dual-task walking versus seated, likely to provide stable spatio-temporal gait mechanics during dual-task walking. This possibility agrees with prior studies suggesting that higher frontal, central and temporal theta power reflect greater engagement of attentional, working memory resources, and sensorimotor integration/processing for error monitoring to collectively ensure successful balance/walking regulation. Such a state would enable the maintenance of adaptive walking mechanics (Aghajan et al. 2017; Hülsdünker et al. 2015; Sipp et al. 2013; Slobounov et al. 2009).

Besides, theta power modulations, low-alpha power was attenuated from seated to walking suggesting greater cortical activity due to enhanced general arousal (Gentili et al.,^ 2018^; Pruziner et al., 2019; Shaw et al., 2018 ). Enhanced engagement of the central, parietal and occipital regions would be needed during dual-task walking versus seated since they have a critical role in motor control, sensorimotor prediction and visual feedback processing for movement regulation, respectively. Also, both low- and high- alpha power were attenuated over the entire scalp from seated to walking. This agrees with the idea that both general arousal and task-specific activity increase with elevated challenge due to cognitive demand or task execution. However, these alpha modulations were observed only for the low and medium demand for seated versus walking, but not for the highest cognitive demand nor for the walking condition when the cognitive demands vary. Possibly under these two latter conditions, the modulation of resources was limited since the participants were somewhat maxed out. These theta and alpha power modulations agree with the elevation of both ratios’ power which suggest enhanced mental workload from seated to dual-task walking as supported by previous similar studies (Gevins and Smith 2003; Pruziner et al. 2019; Shaw et al. 2019a). The greater elevation of FTPA from seated to walking in individuals with transtibial versus transfemoral lower limb loss observed by the exploratory analysis suggests that the former was possibly further driving the elevation of power ratio observed for the entire injured group. Altogether these findings revealed that when switching from seated to dual-task walking both groups recruited several neurocognitive mechanisms, along with a similar/poorer performance, representing attenuated cognitive-motor efficiency (Hatfield et al. 2020; Gentili et al. 2018).

### Specific differences in performance, mental workload, and biomechanical alterations for individuals with and without lower limb loss

#### Effect of the cognitive task demands

Although individuals both with and without limb loss exhibited similar neurocognitive features, an elevation of the cognitive task demands led to reduced low-alpha power in both hemispheres for individuals with but not without lower limb loss across both task conditions. This difference suggests that neural resources related to general arousal increased when individuals with limb loss faced a greater cognitive task demand. Importantly, when considering both these low-alpha power changes and those for the response time performance, individuals with versus without lower-limb loss exhibited lower cognitive-motor efficiency across the various cognitive task demands since walking was maintained, but with greater cortical activity. While an elevation from low to high cognitive demand resulted in a performance decrement (increased response time) for both groups, only individuals with lower limb loss increased the recruitment of neural resources (bilateral reduction of low-alpha power), again resulting in lower cognitive-motor efficiency. Second, while uninjured individuals maintained the recruitment of resources and performance from a low to a medium cognitive demand, the maintenance of performance for individuals with lower limb loss was executed at greater cost since greater resources (reduced low-alpha power in left hemisphere) were engaged. Finally, while the uninjured individuals maintained their performance and resources from a medium to a high cognitive demand, injured individuals exhibited both a performance decrement and an increased recruitment of resources (reduced low-alpha power in the right hemisphere) leading to lower cognitive-motor efficiency. Collectively, these various contrasts suggest that individuals with lower-limb loss exhibited a reduction in cognitive-motor efficiency (decreased/same performance with increased resource recruitment) compared to their uninjured counterparts as the cognitive task demand increased for both the seated and walking conditions.

#### Effect of task execution condition (seated vs. dual-task walking)

A second important difference between the groups was that uninjured individuals revealed a decrease of high-alpha power over the whole scalp from seated to walking whereas the same comparison revealed that such an attenuation was only observed in the central, parietal and occipital regions for individuals with a lower limb loss. This suggests that compared to being seated, uninjured individuals exhibited a broad scalp activity elevation whereas the increase of cortical activity was more limited during dual-task walking among individuals with lower-limb loss. A possible explanation of these specific differences is that uninjured individuals could broadly recruit task-specific resources to perform the concurrent task while walking whereas individuals with lower-limb loss engage a subset of these resources. Possibly, the engagement of the central, parietal and occipital regions for both uninjured and injured individuals would be critical for maintaining the spatio-temporal mechanics during dual-task walking independently of the concurrent task demands as it is well-established that the central, parietal and occipital regions implement neural processes that are critical for dual-task walking (Pruziner et al.,^ 2019^; Shaw et al. 2018, 2019b; Beurskens et al. 2016; Nenna et al.,2021 ). It is reasonable that the central region would be a critical contributor for supraspinal regulation of the gait during dual-task walking in both uninjured and injured individuals based on its central role in motor behavior (Pruziner et al., 2019; Shaw et al. 2018; Beurskens et al. 2016; Nenna et al., 2021 ). This similar activity modulation of the central region in both groups agrees, to some degree, with prior work suggesting that individuals with and without lower limb can reliably activate specific leg muscles, albeit the former was more variable than the latter, and these findings were obtained during walking and not dual-task walking as examined here (Huang et al., 2012). In addition, it was demonstrated that the parietal regions are involved in sensory integration, sensorimotor predictions and action monitoring which are all likely functions to ensure gait stability during dual-task walking (Shaw et al. 2019a, 2019a; Nenna et al.,2021 ; Wilkens et al., 2023; li et al., 2022). Finally, the occipital region ensures visuomotor processing and is particularly important for feedback control of movement regulation (Shaw et al. 2019a, 2019a; Nenna et al.,2021 ). Such regulation would likely be engaged for dual-task walking to make sure that the mechanics (including the pace) are maintained. The exploratory analysis revealed that individuals with transfemoral relative to those with transtibial limb loss may have a more marked reduction of high-alpha power from seated to walking in the cortical regions mentioned above, possibly suggesting the need to further modulate the cortical dynamics to maintain walking due to more extensive compromised limb biomechanics. The absence of modulation in the frontal and temporal activation among individuals with lower limb loss could possibly reflect a lack of engagement of the frontally and temporally-mediated adaptive task-specific processes that are critical for executing the concurrent task while walking. This is consistent with previous studies that have revealed that frontal and/or temporal underactivity would reflect impaired neurocognitive processes (e.g., attention, inhibition, sensory processing/integration) as observed in various neurological pathologies (e.g., patients with ADHD and schizophrenia, Arcuri et al. 2012; Cubillo et al. 2011; Huang et al. 2025; Smith et al. 2006). This could explain why the overall accuracy on the concurrent task was reduced by both the demand and task execution condition (as revealed by the Demand x Condition interaction effect) for injured individuals whereas this was not observed for uninjured individuals (only a main effect of Demand was detected). Also, this result could be related to the elevation of hemispheric driven general arousal as task demands increase observed for injured, but not uninjured, individuals since it may possibly contribute to compensating this absence of frontal and temporal high-alpha modulation. This interpretation of the results is also consistent with the observation that perceived physical demand for the participants with vs. without lower-limb loss revealed a greater (> 1.5 times) effect size for the condition of task execution. The ability for uninjured individuals to deploy their neural resources more broadly and consistently may underlie robust and flexible cognitive-motor control processes, offering a postural reserve large enough to maintain walking mechanics while successfully attending to the concurrent task whereas the limited deployment of such neural resources for the injured individuals achieves stable walking mechanics at the cost of the concurrent task performance.

### Conclusions, limitations, and future work

This study revealed that, when facing increasing cognitive-motor task demands, both individuals with and without lower-limb loss generally maintained walking mechanics and shared common neurocognitive processes underlying a comparable modulation of mental workload. However, an elevation of cognitive task demands led to a lower cognitive-motor efficiency in individuals with versus without lower-limb loss. Importantly, the mental workload modulation can be altered among individuals with lower-limb loss since they are unable to recruit their cognitive-motor resources as consistently and broadly during dual-task walking. Such a limited recruitment of neural resources may explain the lack of robustness and flexibility in their cognitive-motor control, resulting in a lower postural reserve and ultimately leading in further prioritizing the maintenance of (temporal-spatial characteristics of) walking over the concurrent task. Longer term potential application of this work could contribute to providing a comprehensive assessment of both performance and cortical dynamics underlying the mental workload to ultimately gauge cognitive-motor efficiency. Such a comprehensive assessment would allow monitoring the patients’ progress, informing the decision for patients’ release, comparing patients’ status, or assessing prosthesis design. For instance, patients could reveal similar performance with two different prosthesis designs. However, if one prosthesis causes greater cortical activation, indicating a lower cognitive-motor efficiency, then it may need to be revised. There were several limitations to this study. First, the injured group was limited in size and also included a combination of individuals with lower-limb loss at the transtibial and transfemoral level. While this heterogeneity was due to the challenge imposed by recruitment constraints, such a combination is not desirable since the level of limb loss (transtibial versus transfemoral) can affect the walking biomechanics and the neurocognitive processes (Shaw et al. 2019a). The exploratory analysis indicated that most metrics derived from walking mechanics and EEG spectral dynamics in this study appeared to be comparable for both individuals with transtibial and transfemoral lower-limb loss. The only behavioral measure revealing a consistent difference was the smaller stride width observed for individuals with transtibial versus transfemoral lower-limb loss while walking. Also, the high-alpha power decrease and FTFA elevation observed from seated to dual-task walking suggests a modulation having the same directionality for both injured subgroups although these seem more marked for individuals with transfemoral and transtibial lower-limb loss, respectively. Also, the use of a within-subjects design may have limited between-subgroup differences. Thus, while the few differences in walking mechanics and EEG influenced by the level of limb loss agree with prior work, overall, they also allowed confirmation and refinement of the findings of the current study (Shaw et al. 2019b). Second, while providing the ability to self-modulate walking speed is a core feature of the study design and novel contribution to the larger literature, particularly for extended durations of walking that are needed for the neurocognitive outcomes, the underlying control algorithms that modulate treadmill speed do not necessarily fully replicate real-world walking behaviors. In addition, there is always the possible presence of noise in EEG during data collection, particularly with dual-task walking, but several arguments suggest that it was not the main driver for the EEG dynamics. First, the mental workload was examined continuously and not synchronized to the mechanical event (e.g., heel strike) and thus likely limited impact (Kline et al. 2015; Shaw et al. 2019a) while the use of shielded cables has been shown to decrease noise in EEG under such conditions (Kline et al. 2015; Nathan and Contreras-Vidal 2016; Pruziner et al., 2019 ; Reis et al. 2014; Shaw et al. 2019a). The quality of the EEG signals was likely not significantly degraded during walking although a more aggressive denoising led to a decrease in the number of epochs kept for all participants, which was more pronounced in injured individuals and particularly those using a transfemoral prosthesis. Another limitation is that the focus of the study was limited to regional EEG without examining their relationships through functional connectivity. The examination of cortico-cortical communications could inform relevant interregional interactions such as the fronto-parietal network underlying attentional and working memory control, which would be relevant in this study (e.g., greater fronto-parietal theta connectivity would be expected in individuals with versus without lower limb loss; Cavanagh and Frank 2014; Corbetta and Shulman 2002; Sauseng et al. 2005, 2007; Shaw et al. 2019b). Another limitation is that, as it is the case in complex ecologically valid tasks, the cortical dynamics observed here were likely driven by a combination of cognitive, sensory and motor processes, which could not be necessarily fully disentangled (although it was not the goal of this study) and as such future work would be needed to investigate the different contributions of each mechanism. Future work should include a population of injured individuals large enough to examine separately the influence of the level of limb loss as well as the type of prosthesis in combination with a more comprehensive examination of the brain dynamics to include functional connectivity.

## Supplementary Information

Below is the link to the electronic supplementary material.

Supplementary Material 1

Supplementary Material 1 - revised

## Data Availability

The data set for this manuscript is not publicly available.
