# Supplementary material for: Using a self-modulated treadmill as a novel approach to study cognitive-motor and biomechanical outcomes during dual-task walking in individuals with and without lower limb loss
Source: Exp Brain Res. 2026 Jan 21;244(2):30. doi: 10.1007/s00221-025-07209-2 (PMC12823759; doi:10.1007/s00221-025-07209-2)
Supplement: Supplementary file 2 — Supplementary Material 1 - revised [file 221_2025_7209_MOESM2_ESM.pdf]

Supplementary Material

1. Results descriptive statistics

1.1 Biomechanics

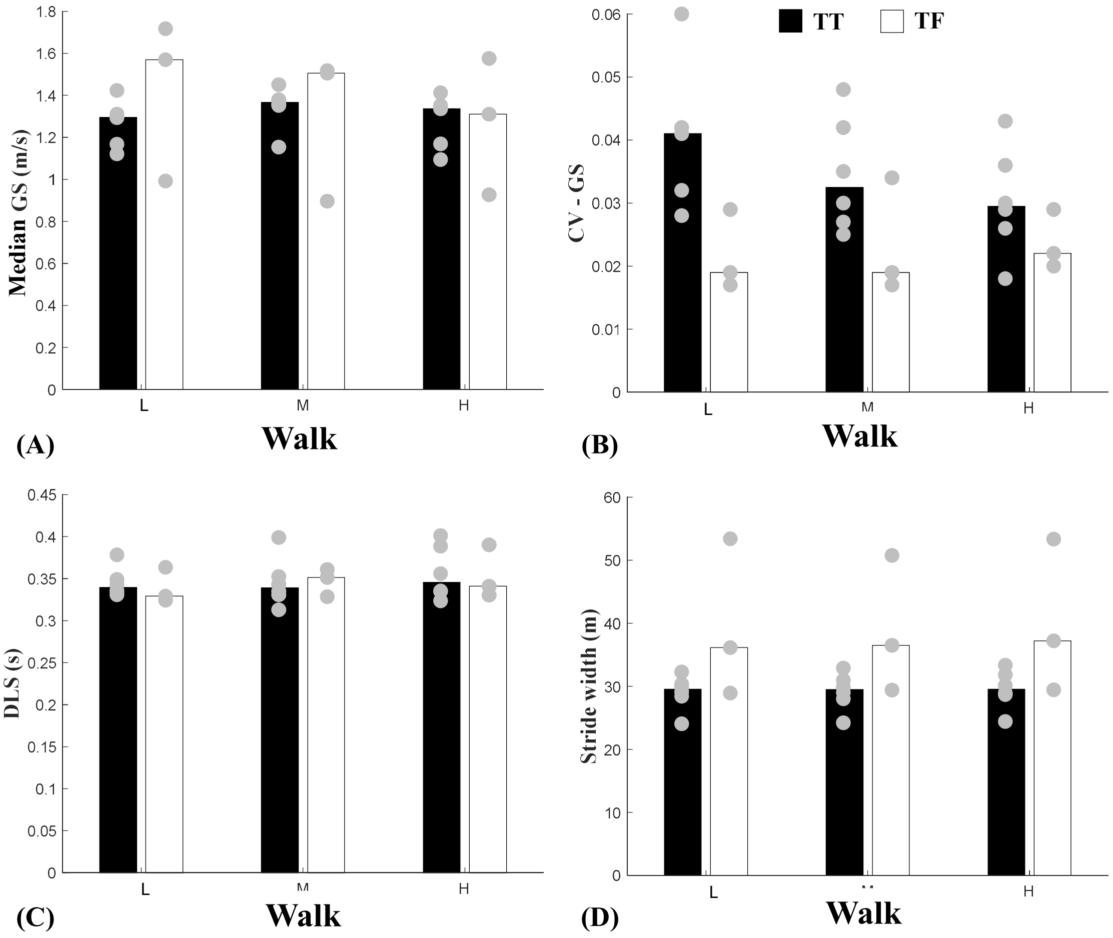

Figure S1. Walking mechanics measures. Median GS, CV, DLS and stride width for individuals with transtibial (TT, black bars) and transfemoral (TF, white bars) limb loss when performing the cognitive task under and low (L), medium (M) and high (H) level of demand. The gray fill circles represent the individuals' data points.

Table S1. Median [Low quartile High quartile] for walking mechanics in individuals with transtibial (TT) and transfemoral (TF) lower limb loss during dual-task walking under low (L), medium (M) and high (H) cognitive demands. GS: gait speed; CV: coefficient of variation of GS; DLS" double limb support.

	**WALK**					
	**TT LL**			**TF LL**		
	**L**	**M**	**H**	**L**	**M**	**H**
**GS**	1.30 [1.20 1.31]	1.37 [1.35 1.38]	1.34 [1.21 1.35]	1.57 [1.28 1.64]	1.50 [1.20 1.51]	1.31 [1.12 1.44]
**CV**	0.04 [0.03 0.04]	0.03 [0.03 0.04]	0.03 [0.03 0.03]	0.02 [0.02 0.02]	0.02 [0.02 0.03]	0.02 [0.03 0.03]
**DLS**	0.34 [0.33 0.35]	0.34 [0.33 0.35]	0.35 [0.33 0.38]	0.33 [0.33 0.35]	0.33 [0.34 0.36]	0.33 [0.34 0.37]
**Stride width**	29.55 [28.62 30.24]	29.47 [28.27 30.71]	29.54 [28.76 31.41]	36.13 [32.52 44.78]	36.51 [32.96 43.65]	37.23 [33.34 45.30]

1.2 Cognitive performance

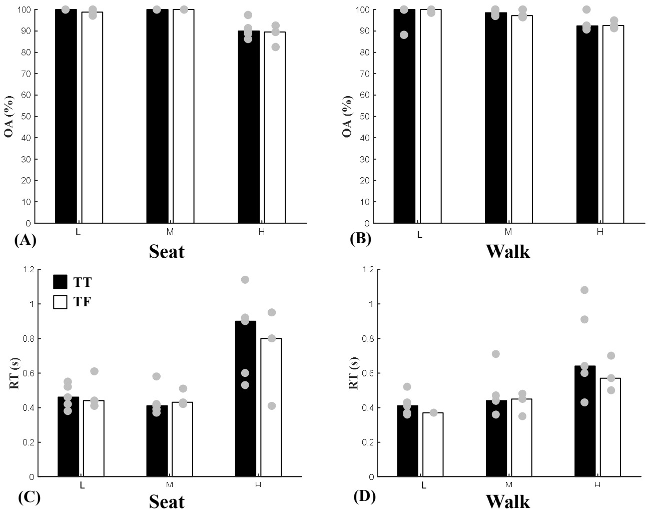

Figure S2. Cognitive performance in individuals with transtibial (TT, black bars) and transfemoral (TF, white bars) limb loss when seated (left column) and dual-task walking (right column) under a low (L), medium (M) and high (H) demand. The gray fill circles represent the individuals' data points. OA: Overall Accuracy; RT: Response Time.

Table S2. Median [Low quartile High quartile] for cognitive performance in individuals with transtibial (TT) and transfemoral (TF) limb loss when seated and dual-task walking under a low (L), medium (M) and high (H) demand. OA: Overall Accuracy; RT: Response Time.

	SEAT					
	TT			TF		
	L	M	H	L	M	H
**OA**	100.00 [100.00 100.00]	100.00 [100.00 100.00]	90.00 [88.75 91.25]	98.75 [97.95 99.38]	100.00 [100.00 100.00]	89.47 [85.99 90.99]
**RT**	0.46 [0.42 0.52]	0.41 [0.39 0.42]	0.90 [0.60 0.92]	0.44 [0.43 0.53]	0.43 [0.42 0.47]	0.80 [0.60 0.88]

	WALK					
	TT			TF		
	L	M	H	L	M	H
**OA**	100.00 [100.00 100.00]	98.48 [97.40 100.00]	92.31 [92.00 92.50]	100.00 [99.28 100.00]	97.18 [96.78 98.59]	92.50 [91.88 93.69]
**RT**	0.41 [0.37 0.43]	0.44 [0.44 0.47]	0.64 [0.60 0.91]	0.37 [0.37 0.37]	0.45 [0.40 0.47]	0.57 [0.53 0.64]

1.3 Theta power

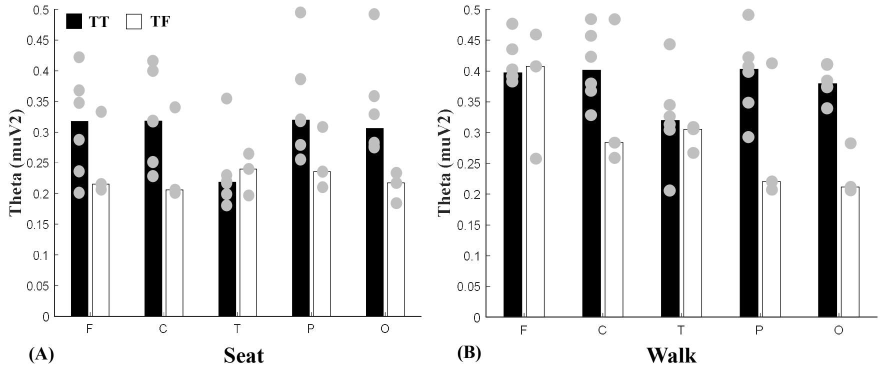

Figure S3. Changes in median theta power in the frontal (F), central (C), temporal (T), parietal (P) and occipital (O) region in individuals with transtibial (TT, black bars) and transfemoral (TF, white bars) limb loss when seated (left column) and walking (right column) when performing the cognitive task under an easy (E), medium (M) and high (H) level of demand. The gray fill circles represent the individuals' data points.

Table S3. Median [Low quartile High quartile] for theta power in the frontal (F), central (C), temporal (T), parietal (P) and occipital (O) regions in individuals with transtibial (TT) and transfemoral (TF) limb loss when seated and dual-task walking.

	**SEAT**									
	**TT**					**TF**				
	**F**	**C**	**T**	**P**	**O**	**F**	**C**	**T**	**P**	**O**
**Theta**	0.32 [0.25 0.36]	0.32 [0.27 0.38]	0.22 [0.20 0.23]	0.32 [0.29 0.37]	0.31 [0.28 0.35]	0.22 [0.21 0.27]	0.21 [0.20 0.27]	0.24 [0.22 0.25]	0.24 [0.22 0.27]	0.22 [0.20 0.23]

	**WALK**									
	**TT**					**TF**				
	**F**	**C**	**T**	**P**	**O**	**F**	**C**	**T**	**P**	**O**
**Theta**	0.40 [0.39 0.43]	0.40 [0.37 0.45]	0.32 [0.31 0.34]	0.40 [0.36 0.42]	0.38 [0.37 0.40]	0.41 [0.33 0.43]	0.28 [0.27 0.38]	0.31 [0.29 0.31]	0.22 [0.21 0.32]	0.21 [0.21 0.25]

1.4 Low-alpha power

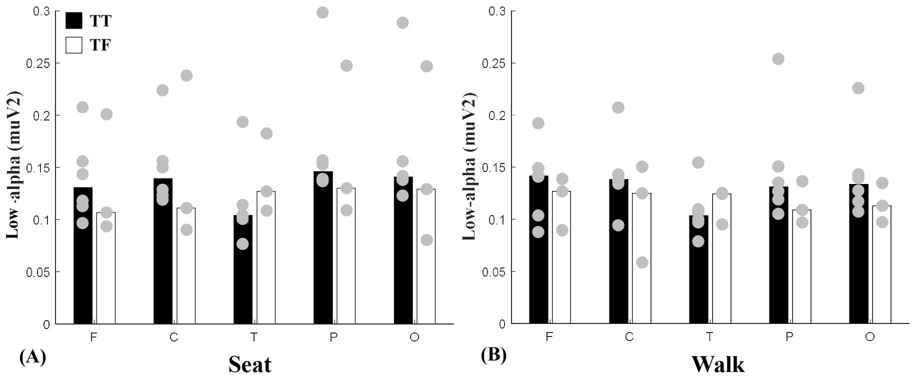

Figure S4. Changes in median low-alpha power in the frontal (F), central (C), temporal (T), parietal (P) and occipital (O) region in individuals with transtibial (TT, black bars) and transfemoral (TF, white bars) limb loss when seated (left column) and walking (right column) when performing the secondary cognitive task under an easy (E), medium (M) and high (H) level of demand. The gray fill circles represent the individuals' data points.

Table S4. Median [Low quartile High quartile] for low-alpha power in the frontal (F), central (C), temporal (T), parietal (P) and occipital (O) regions in individuals with transtibial (TT) and transfemoral (TF) limb loss when seated and dual-task walking.

	**SEAT**									
	**TT**					**TF**				
	**F**	**C**	**T**	**P**	**O**	**F**	**C**	**T**	**P**	**O**
**Low-alpha**	0.13 [0.11 0.15]	0.14 [0.12 0.15]	0.10 [0.10 0.11]	0.15 [0.14 0.16]	0.14 [0.14 0.15]	0.11 [0.10 0.15]	0.12 [0.10 0.17]	0.10 [0.22 0.15]	0.13 [0.12 0.27]	0.13 [0.10 0.19]

	**WALK**									
	**TT**					**TF**				
	**F**	**C**	**T**	**P**	**O**	**F**	**C**	**T**	**P**	**O**
**Low-alpha**	0.14 [0.11 0.15]	0.14 [0.14 0.14]	0.10 [0.10 0.44]	0.13 [0.12 0.15]	0.13 [0.12 0.14]	0.13 [0.11 0.13]	0.13 [0.09 0.14]	0.12 [0.11 0.12]	0.11 [0.10 0.12]	0.11 [0.11 0.12]

1.5 High-alpha power

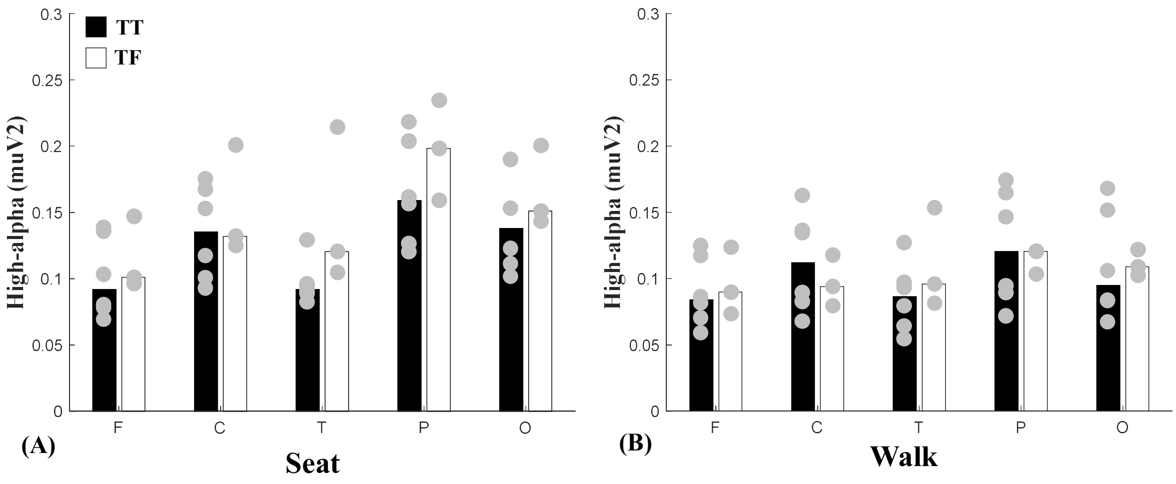

Figure S5. Changes in median high-alpha power in the frontal (F), central (C), temporal (T), parietal (P) and occipital (O) region in individuals with transtibial (TT) and transfemoral (TF) limb loss when seated (left column) and walking (right column) when performing the secondary cognitive task under an easy (E), medium (M) and high (H) level of demand. The gray fill circles represent the individuals' data points.

Table S5. Median [Low quartile High quartile] for high-alpha power in the frontal (F), central (C), temporal (T), parietal (P) and occipital (O) regions in individuals with transtibial (TT, black bars) and transfemoral (TF, white bars) limb loss when seated and dual-task walking.

	SEAT									
	TT					TF				
	F	C	T	P	O	F	C	T	P	O
**High-alpha**	0.09 [0.08 0.13]	0.14 [0.11 0.16]	0.09 [0.09 0.10]	0.16 [0.13 0.19]	0.14 [0.11 0.18]	0.10 [0.10 0.12]	0.13 [0.13 0.17]	0.12 [0.11 0.17]	0.20 [0.18 0.22]	0.15 [0.15 0.18]

	WALK									
	TT					TF				
	F	C	T	P	O	F	C	T	P	O
**High-alpha**	0.08 [0.07 0.11]	0.11 [0.08 0.14]	0.09 [0.07 0.10]	0.12 [0.09 0.165]	0.09 [0.08 0.14]	0.09 [0.08 0.11]	0.09 [0.09 0.11]	0.10 [0.09 0.12]	0.12 [0.11 0.12]	0.11 [0.11 0.12]

1.6 Power ratios

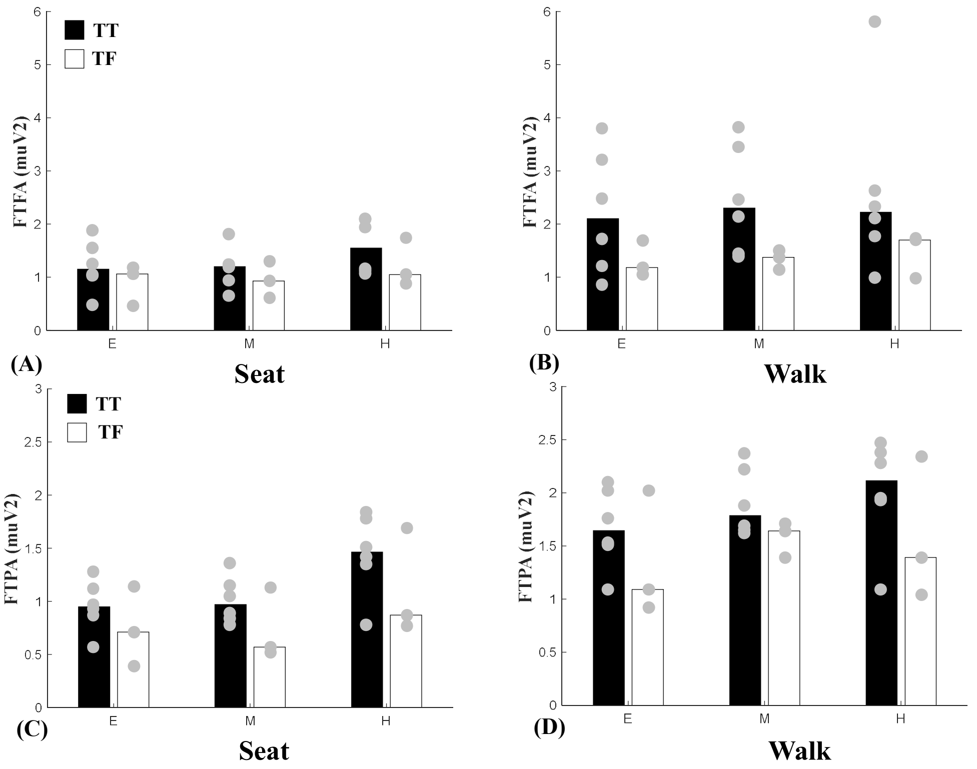

Figure S6. Changes in median FTFA and FTPA ratio power in individuals with transtibial (TT, black bars) and transfemoral (TF, white bars) limb loss when seated (left column) and walking (right column) when performing the secondary cognitive task under an easy (E), medium (M) and high (H) level of demand. The gray fill circles represent the individuals' data points.

Table S6. Median [Low quartile High quartile] for FTFA and FTPA ratio power in individuals with transtibial (TT, black bars) and transfemoral (TF, white bars) limb loss when seated and dual-task walking under a low (L), medium (M) and high (H) demand.

	SEAT					
	TT			TF		
	L	M	H	L	M	H
FTFA	1.15 [1.03 1.47]	1.20 [1.00 1.24]	1.55 [1.13 2.05]	1.06 [0.76 1.12]	0.93 [0.77 1.12]	1.05 [0.97 1.39]
FTPA	0.95 [0.88 1.08]	0.97 [0.85 1.13]	1.47 [1.37 1.71]	0.71 [0.55 0.92]	0.57 [0.55 0.92]	0.87 [0.81 1.28]

	WALK					
	TT			TF		
	L	M	H	L	M	H
FTFA	2.10 [1.34 3.02]	2.30 [1.62 3.20]	2.22 [1.86 2.56]	1.18 [1.11 1.43]	1.37 [1.25 1.43]	1.70 [1.34 1.72]
FTPA	1.64 [1.51 1.96]	1.78 [1.66 2.14]	2.11 [1.93 2.35]	1.09 [1.01 1.55]	1.64 [1.52 1.67]	1.39 [1.22 1.87]

2/ Results NASA-TLX survey

For the other dimensions of the NASA TLX, there was an effect of task Demand on the perceived temporal demand ($F(1.341, 18.779) = 12.176$, $p = 0.001$, $\eta_P^2 = 0.465$), performance ($F(1.073, 15.021) = 7.050$, $p = 0.017$, $\eta_P^2 = 0.335$), effort ($F(1.120, 15.678) = 25.279$, $p < 0.001$, $\eta_P^2 = 0.644$), and frustration ($F(1.178, 16.497) = 7.678$, $p = 0.011$, $\eta_P^2 = 0.354$); participants perceived a diminished performance along with an elevation of the temporal demand, effort, and frustration from low to high demand and from medium to high demand (low vs. high: $p < 0.006$, $0.936 \leq d \leq 1.498$; medium vs. high: $p < 0.028$, $0.756 \leq d \leq 1.189$ for all comparisons considered).
